# Comparison of clinical features and 3-month treatment response among three different choroidal thickness groups in polypoidal choroidal vasculopathy

**DOI:** 10.1371/journal.pone.0184058

**Published:** 2017-09-08

**Authors:** Mingui Kong, Sung Min Kim, Don-Il Ham

**Affiliations:** 1 Hangil Eye Hospital, Incheon, Korea; 2 Department of Ophthalmology, Catholic Kwandong University College of Medicine, Incheon, Korea; 3 Department of Ophthalmology, Samsung Medical Center, Sungkyunkwan University School of Medicine, Seoul, Korea; Massachusetts Eye & Ear Infirmary, Harvard Medical School, UNITED STATES

## Abstract

Eyes with polypoidal choroidal vasculopathy (PCV) were recently reported to have various choroidal thickness, and choroidal thickness might be associated with visual outcome in the treatment of many retinal disorders. The range of subfoveal choroidal thickness (SFCT), clinical features, and 3-month treatment response among three groups having different range of SFCT were investigated in PCV eyes. In 78 treatment-naïve eyes with PCV, SFCT was measured using optical coherence tomography. Eyes were classified into thin, medium, and thick groups, using mean and one standard deviation of SFCT. Clinical features and imaging findings were compared among the three groups. Some eyes were treated with three consecutive monthly injection of anti-vascular endothelial growth factor (VEGF) as an initial treatment. They were also classified into three thickness groups, and the short-term post-treatment improvement in visual acuity and central retinal thickness were compared among groups. The mean SFCT was 271.9 ± 135.6 μm. Twelve, 53, and 13 eyes were classified into thin (<136.3 μm), medium (136.3–407.5 μm), and thick (>407.5 μm) groups, respectively. The thin group showed older age, lower visual acuity, and a higher prevalence of fundus tessellation than the other two groups (P <0.05). In multiple linear regression analyses, baseline BCVA was correlated with baseline SFCT. Forty-six eyes completed three consecutive anti-VEGF treatments. The thin group showed no visual improvement after treatment (P = 0.141), unlike the other two groups showing visual improvement (P<0.05). Eyes with PCV have a broad range of SFCT, and PCV eyes with a thin choroid manifest worse visual function than eyes with a medium or thick choroid.

## Introduction

Polypoidal choroidal vasculopathy (PCV) is a common retinal disease in Asians, characterized by abnormal vascular channels and polypoidal vascular dilatation which could be observed on indocyanine green angiography (ICGA).[1,2] Although about half of patients with PCV will have a favorable natural course, the remaining half lose their vision due to recurrent bleeding and persistent exudation.[3] Unfortunately, anti-vascular endothelial growth factor (VEGF) treatment is less useful for patients with PCV than in those with typical age-related macular degeneration (AMD). Other treatment modalities, including photodynamic therapy (PDT) or combination therapy are commonly used to treat PCV, and several studies have attempted to classify PCV to identify subgroups with better or worse treatment outcomes.[4–10]

Although the choroid is generally thicker in eyes with PCV than in those with typical AMD and healthy controls,[11,12] several studies have shown that many eyes with PCV have a thin to medium-thickness choroid.[13,14] Furthermore, SFCT was associated with anatomic and functional outcome after intravitreal anti-VEGF injection treatment in patients with retinal disorders, including AMD and diabetic maculopathy.[15,16] Thus, eyes with PCV probably have a various SFCT and may have different clinical features according to the SFCT.

The purpose of this study was to investigate the range of SFCT in PCV eyes, and to compare clinical features, including short-term treatment response to anti-VEGF treatment, among three different SFCT-range groups of patients with PCV.

### Subjects and methods

We retrospectively reviewed the medical records of patients diagnosed with PCV at Samsung Medical Center, Seoul, Korea, from November 2009 to February 2015. All investigations adhered to the tenets of the Declaration of Helsinki, and this study was approved by the institutional review board and ethics committee at Samsung Medical Center.

All patients had undergone ocular examinations including best corrected visual acuity (BCVA), a slit lamp examination, and imaging tests including color fundus photography, enhanced depth imaging optical coherent tomography (EDI OCT), fluorescent angiography (FA), and ICGA. Fundus color photographs were taken with a Topcon camera (IX50, Topcon, Paramus, NJ, USA). EDI OCT, FA, and ICGA were performed using a Spectralis HRA+OCT instrument (Spectralis HRA+OCT; Heidelberg Engineering, Heidelberg, Germany).

PCV was diagnosed based on the presence of polypoidal vessel dilation with abnormal vascular channels on ICGA using a confocal scanning laser ophthalmoscope. Inclusion criteria for this study were age of patients ≥ 50 years old, and symptomatic PCV in the macular area with no previous treatment. Only the right eye was included if both eyes had PCV, to exclude any possible influence resulting from including both eyes from the same patient. Exclusion criteria were choroidal neovascularization (CNV) with AMD, retinal angiomatous proliferation (RAP), a large abnormal vascular network across the major vascular arcade, polypoidal vessels developed from initial neovascular AMD, geographic atrophy, massive submacular hemorrhage or fibrosis, pathologic myopia (spherical equivalent ≤ -6 diopters or axial length ≥ 26mm), glaucoma, uveitis, diabetic retinopathy, retinal vascular occlusion, vitreomacular traction, central serous chorioretinopathy, history of trauma or vitreoretinal surgery, and history of cataract surgery within 1 year.

SFCT was measured manually at the foveal center, from the outer portion of the hyper-reflective line corresponding to the retinal pigment epithelium (RPE) to the inner surface of the sclera using software supplied with the SD OCT device. All measurements were made by two observers (M.K. and S.M.K.), and mean values were used in the analysis. Central retinal thickness (CRT) was measured using the Spectralis built-in software. Topographic retinal thickness maps were produced in nine subfields of the Early Treatment Diabetic Retinopathy Study grid, and the value of central inner circle was used as CRT. The presence of choroidal vascular hyperpermeability was assessed using ICGA. Choroidal vascular hyperpermeability was defined as multifocal areas of hyperfluorescence with blurred margins within the choroid in the late phase of ICGA.[17] The presence of fundus tessellation and soft drusen was assessed using fundus color photography of macula. Fundus tessellation was defined as variation in visibility of the large choroidal vessels, and grade 2 or more was counted as fundus tessellation.[18] Soft drusen were considered to be present, if extensive (>15) small drusen (<63 μm), or more than one intermediate drusen (≥63 μm and <125 μm) or more than one large drusen (≥125 μm) were observed on fundus color photographs.[19] These assessments were performed by two observers (M.K. and S.M.K.) who were blinded to the EDI OCT images. If there was a discrepancy, the senior observer (D.-I.H.) made the final decision.

The eyes were divided into three groups of thin, medium, and thick choroids, based on the SFCT data of total study eyes. A thin choroid was defined as a choroid with SFCT < mean SFCT minus one standard deviation (SD). A thick choroid was defined as a choroid with SFCT > mean SFCT plus one SD. A medium choroid was defined as a choroid with SFCT between the two cut-off values. Age, sex, spherical equivalent (SE) of refractive error, CRT, SFCT, choroidal vascular hyperpermeability, fundus tessellation and soft drusen, were compared among three groups.

To evaluate the short-term treatment response, eyes that completed the initial 3-month treatment with bevacizumab (Avastin; Genentech, South San Francisco, CA, USA) or ranibizumab (Lucentis; Genentech,) were analyzed. Patients underwent three monthly intravitreal injections of 0.05 ml bevacizumab (1.25mg) or three monthly intravitreal injections of 0.05 ml ranibizumab (0.5mg). If an eye was treated with more than one kind of drug or more than one therapeutic method, such as anti-VEGF therapy combined with PDT, the eye was excluded from the treatment response analysis. Treatment response was assessed by the evaluation of BCVA and CRT at baseline and at 3-month follow-up (one month after last anti-VEGF injection).

Data were analyzed using the Kruskall Wallis test, Mann–Whitney test, the chi-square test, Fisher’s exact test and the Wilcoxon signed-ranks test. Pearson’s correlation coefficient test and multiple linear regression with backward stepwise method were conducted to find out factors affecting the visual function. A *P-*value < 0.05 was considered to be statistically significant. All statistical analyses were performed using SPSS 18.0 software (SPSS Inc., Chicago, IL, USA).

## Results

**Fig 1:**
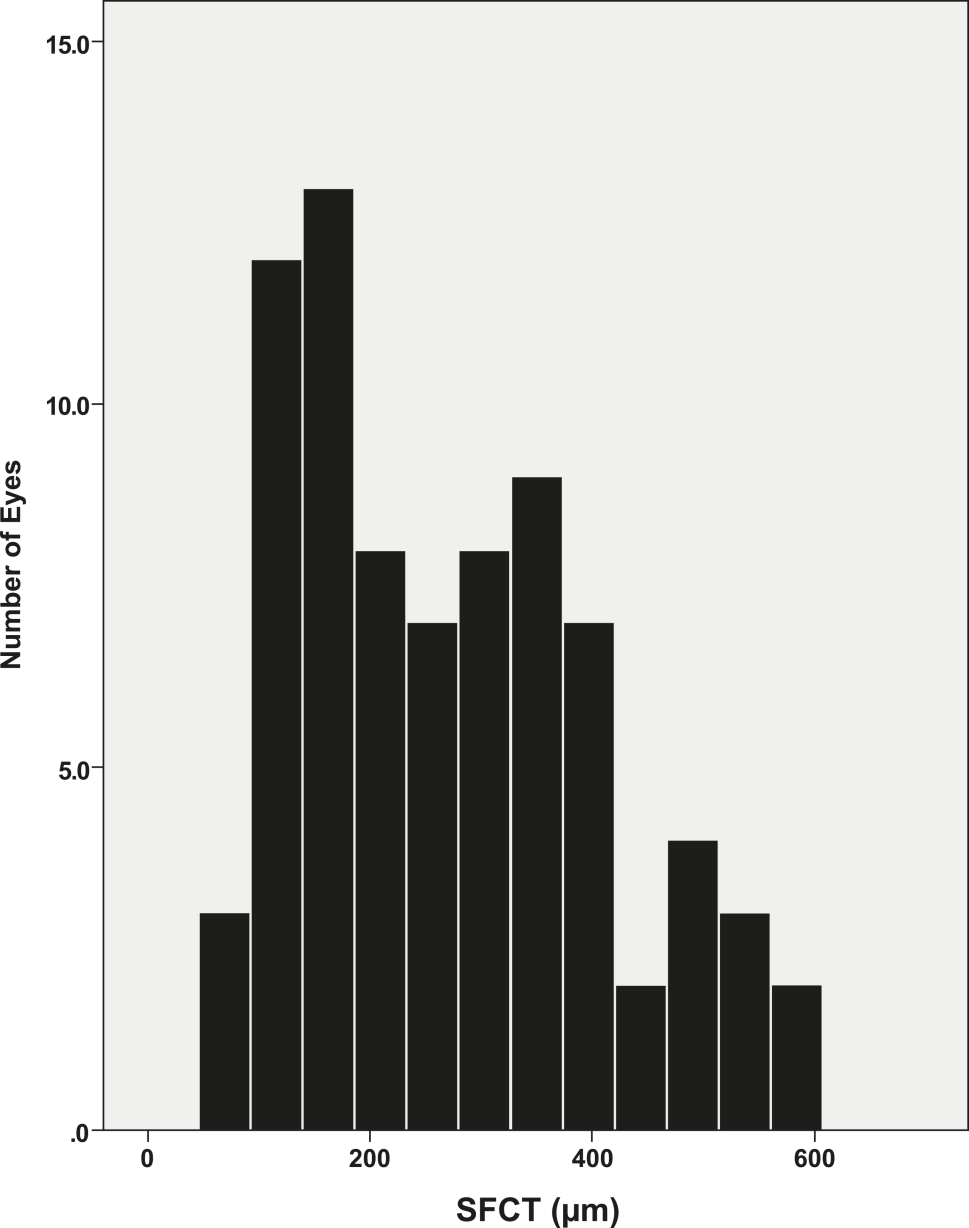
Distribution profile of subfoveal choroidal thickness in 78 eyes with polypoidal choroidal vasculopathy. SFCT: subfoveal choroidal thickness.

Seventy eight eyes in 78 patients were included in this study. Three patients had bilateral PCV, and only right eye was used. Mean age was 70.7 ± 8.6 years (range, 53–91 years), and mean SE of refractive error was 0.52 ± 1.21 diopter (range, -2.13, +3.5 diopter). SFCT was measured, and the range of SFCT was 60–602 μm. Mean SFCT was 271.9 μm (Median SFCT = 258.5 μm) and one standard deviation was 135.6 μm. The distribution profile of measured SFCT was shown in Fig 1.

**Fig 2:**
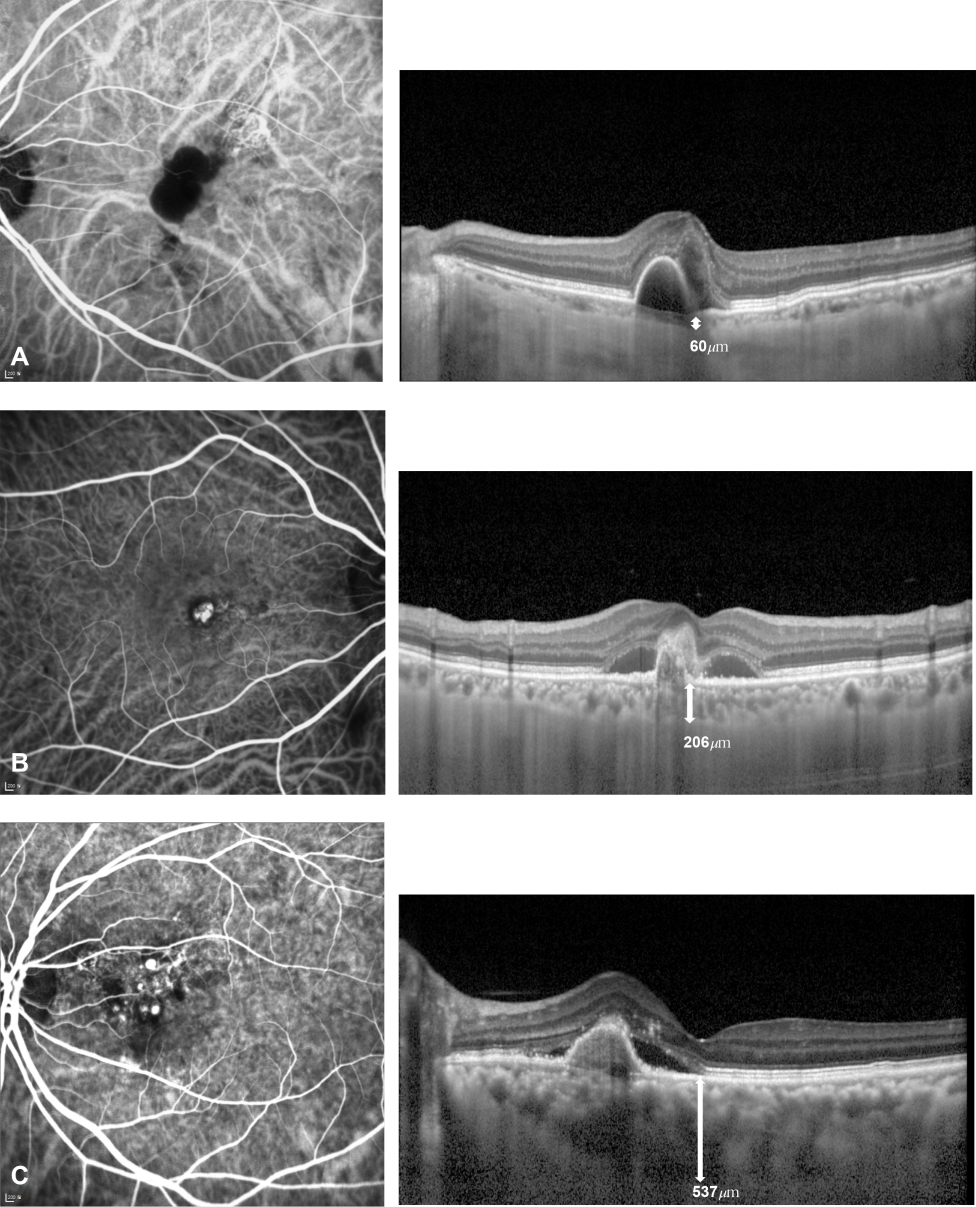
Indocyanine green angiography (ICGA) and enhanced depth imaging optical coherent tomography (EDI OCT) images from the three thickness groups. (A) Left eye of a 78 year old man in the thin group. (B) Right eye of a 70 year old man in the medium group. (C) Left eye of a 61 year old woman in the thick group. Abnormal vascular channels and polyps were seen on ICGA images, and white arrows and figures on EDI OCT images indicate measured subfoveal choroidal thickness.

The two cut-off values for classifying study eyes into the three SFCT groups were 136.3 and 407.5 μm, using mean SFCT ± 1 SD. Thus, eyes with SFCT < 136.3 μm were classified as the thin group, and eyes with SFCT > 407.5 μm were classified as the thick group. The medium group had SFCT values of 136.3–407.5 μm (Fig 2).

### Clinical features among three groups

Demographics and clinical features of three thickness groups were summarized in Table 1.

**Table 1:** Demographics and clinical features of PCV eyes in 3 choroidal thickness groups.

	Thin	Medium	Thick	P-value*	P-value^†^	P-value^‡^	P-value**	Total
Number	12	53	13	N/A	N/A	N/A	N/A	78
SFCT (μm)	107.8 ± 23.6(60–136)	254.0 ± 86.1(137–403)	496.0 ± 56.5(414–602)	N/A	N/A	N/A	N/A	271.9 ± 135.6(60–602)
Age (years)	77.2 ± 8.1(61–91)	71.1 ± 7.9(55–87)	63.5 ± 6.1(53–72)	0.001	0.022	<0.001	0.002	70.7 ± 8.6(53–91)
SE (D)	0.02 ± 1.1(-1, +2.63)	0.64 ± 1.29(-2.13, +3.5)	0.49 ± 0.82(-1.0, +2.25)	0.280	N/A	N/A	N/A	0.52 ± 1.21(-2.13, +3.5)
BCVA (LogMAR)^††^	0.81 ± 0.49(0.22–2.0)	0.51 ± 0.33(0–1.52)	0.44 ± 0.33(0–1.0)	0.023	0.013	0.041	0.481	0.55 ± 0.38(0–2.0)
CRT (μm)	553.8 ± 237.3	407.8 ± 192.8	455.4 ± 184.5	0.082	N/A	N/A	N/A	438.2 ± 205.8
Soft drusen	4 (33.3%)	13 (24.5%)	2 (15.4%)	0.579	N/A	N/A	N/A	19 (24.4%)
Fundus tessellation	12 (100%)	22 (41.5%)	0 (0%)	<0.001	<0.001	<0.001	0.003	34 (43.6%)
Hyper-permeability^‡‡^	7 (58.3%)	40 (75.4%)	12 (92.3%)	0.141	N/A	N/A	N/A	59 (75.6%)

SFCT: subfoveal choroidal thickness; SE: spherical equivalent; BCVA: best corrected visual acuity; CRT: central retinal thickness

* among three groups

^†^ thin vs medium

^‡^ thin vs thick

** medium vs thick

^††^ BCVA at baseline

^‡‡^ hyperpermeability on late phase during indocyanine green angiography

**Fig 3:**
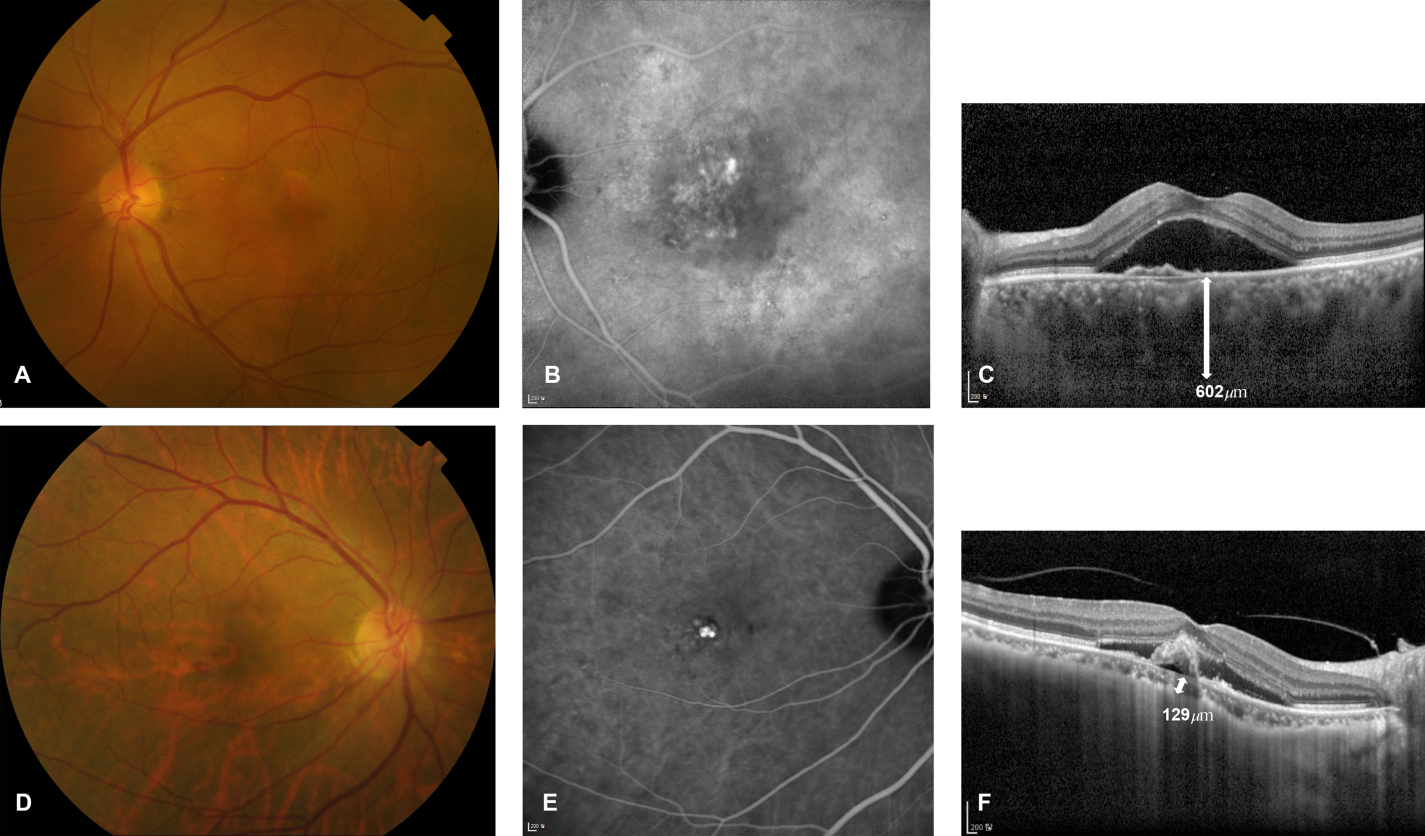
Imaging features of eyes having thick or thin choroid. (A-C) Left eye of a 66 year old man in the thick group. (A) Color photograph showed no fundus tessellation, and (B) late phase indocyanine green angiography (ICGA) image showed hyperfluorescent areas indicating the presence of choroidal hyperpermeability. (C) Subfoveal choroid was thick (white arrow) on enhanced depth imaging optical coherence tomography (EDI OCT) image. (D-F) Right eye of a 70 year old woman in the thin group. (D) Color photograph showed fundus tessellation, and (E) late phase ICGA image revealed the absence of choroidal hyperpermeability. (F) Subfoveal choroid was thin (white arrow) on EDI OCT image.

Twelve, 53, and 13 eyes were classified into the thin, medium, and thick groups. Mean SFCT was 107.8 ± 23.6 μm (range 60–136 μm), 254.0 ± 86.1 μm (range 137–403 μm), 496.0 ± 56.5 μm (range 414–602 μm) in the thin, medium, and thick group, respectively. The thin group was older than the other two groups (P = 0.022 for medium group and P<0.001 for thick group), and the medium group was also older than the thick group (P = 0.002). Refractive error, compared using SE, was not significantly different among groups (P = 0.280). The thin group had worse BCVA than the other two groups (P = 0.013 for medium group and P = 0.041 for thick group). CRT was not different among groups (P = 0.082). Nineteen eyes (24.4%) had soft drusen. The prevalence of soft drusen was not different among three groups (P = 0.579). Fundus tessellation was present in all eyes in the thin group, and was observed more frequently in the thin group than the other two groups (P<0.001). Eight eyes (66.7%) of thin group had fundus tessellation and SFCT less than 125 μm, which is the suggested cut-off value for age-related choroidal atrophy.[20] The prevalence of choroidal vascular hyperpermeability was not different among three groups (P = 0.141) (Fig 3).

### Short-term response to anti-VEGF treatment

Of the 78 eyes, 46 eyes completed three consecutive anti-VEGF treatments as an initial therapy. Eight, 31, and 7 eyes were classified into the thin, medium, and thick groups. Baseline demographics and clinical features of treated eyes were summarized in Table 2.

**Table 2:** Baseline characteristics of 46 eyes treated with anti-vascular endothelial growth factor.

	Thin	Medium	Thick	P-value*	P-value^†^	P-value^‡^	P-value**	Total
Number	8	31	7	N/A	N/A	N/A	N/A	46
SFCT (μm)	106.3 ± 27.3(60–136)	242.1 ± 88.2(137–395)	496.0 ± 54.7(414–588)	N/A	N/A	N/A	N/A	257.1 ± 136.5(60–588)
Age (years)	78.9 ± 7.8(66–91)	71.0 ± 8.2(55–85)	62.9 ± 7.2(53–71)	0.008	0.033	0.006	0.036	71.2 ±9.2(53–91)
SE (D)	0.00 ± 1.26(-1.0, +2.63)	0.50 ± 1.31(-2.13, +3.5)	0.32 ± 0.95(-1.0, +2.25)	0.366	N/A	N/A	N/A	0.38 ± 1.27(-2.13, +3.5)
BCVA (LogMAR)	0.88 ± 0.57(0.22–2.0)	0.57 ± 0.35(0–1.52)	0.49 ± 0.40(0–1.0)	0.319	N/A	N/A	N/A	0.61 ± 0.42(0–2.0)
CRT (μm)	646.6 ± 240.0	426.3 ± 178.1	412.4 ± 140.7	0.065	N/A	N/A	N/A	462.5 ± 203.8

SE: spherical equivalent; SFCT: subfoveal choroidal thickness; CRT: central retinal thickness; BCVA: best corrected visual acuity

* among three groups

^†^ thin vs medium

^‡^ thin vs thick

** medium vs thick

The thin group was older than the other two groups (P ≤ 0.033). No significant difference in refractive error, pre-treatment BCVA and CRT was detected among the three groups (P = 0.366, 0.319, and 0.065, respectively). The analysis of treatment response was summarized in Table 3.

**Table 3:** Visual and anatomic outcomes after short-term treatment with anti-vascular endothelial growth factors (VEGF) in 46 treated eyes.

		BCVA before* (LogMAR)	BCVA after^†^ (LogMAR)	P-value^‡^	CRT before* (μm)	CRT after^†^ (μm)	P-value^‡^
Anti-VEGF treatment (46 eyes)	Thin	0.84 ± 0.50 (0.22–2.0)	0.72 ± 0.35 (0.30–1.40)	0.141	571.1 ± 242.4 (298–984)	317.0 ± 132.4 (205–695)	0.006
	Medium	0.53 ± 0.37(0–1.52)	0.41 ± 0.44 (0–2)	0.008	430.2 ± 181.7(200–938)	287.6 ± 101.6(178–632)	<0.001
	Thick	0.57 ± 0.38 (0.15–1.0)	0.21 ± 0.16 (0–0.52)	0.043	419.7 ± 150.7(237–606)	267.7 ± 82.3 (171–428)	0.046

BCVA: best corrected visual acuity; CRT: central retinal thickness

* before treatment

^†^ BCVA at 1 month after the three consecutive anti-VEFG injections

^‡^ Wilcoxon signed ranks test

**Fig 4:**
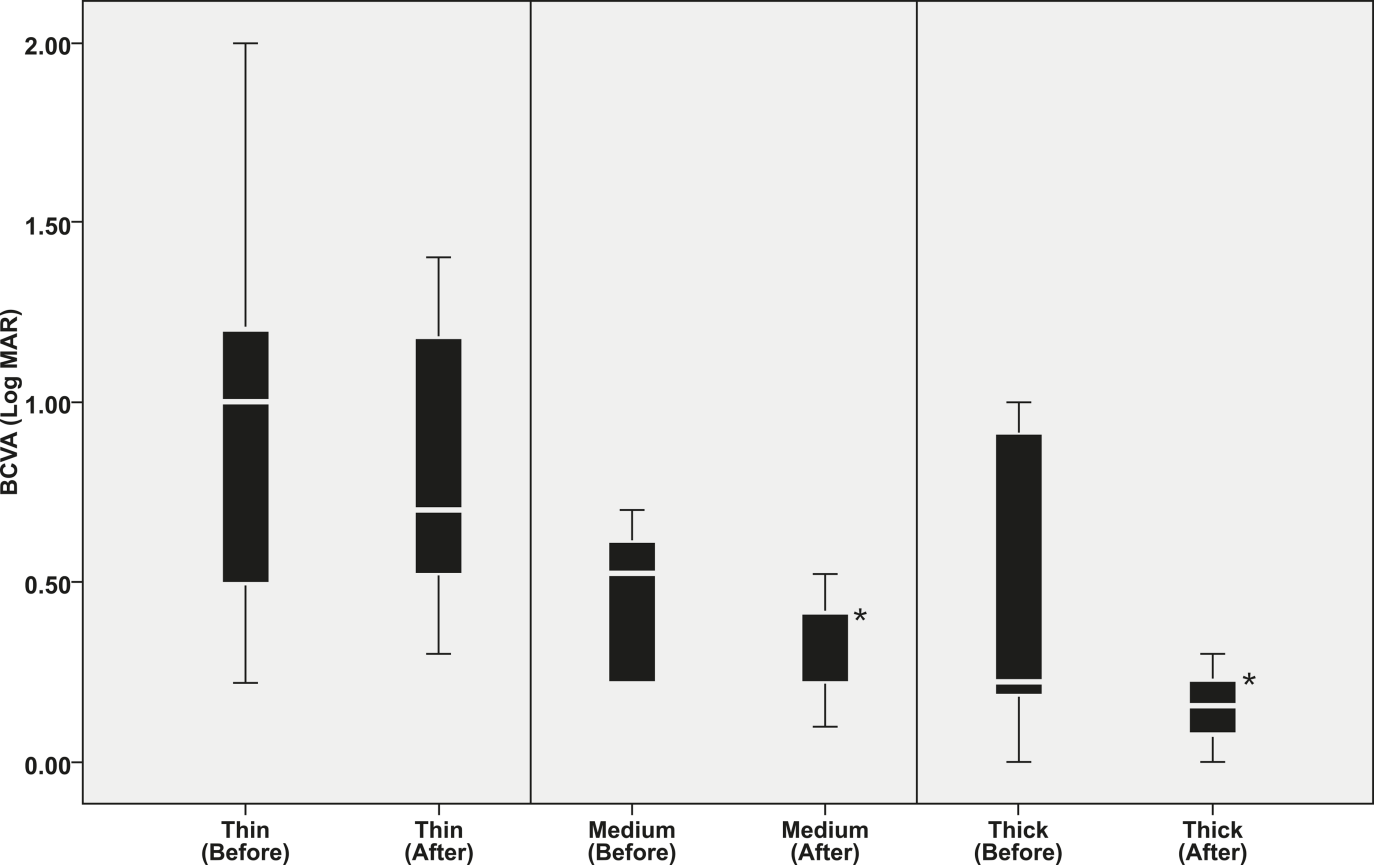
Treatment response in 3 thickness groups. Boxplots showing the distribution of best corrected visual acuity at baseline (before) and at 1 month after 3 consecutive monthly anti-vascular endothelial growth factor treatments (after). The medium and thick groups showed significant visual improvement after treatment (asterisk). However, the thin group showed no significant improvement. BCVA: best corrected visual acuity.

Although the thick and medium groups showed significant visual improvement after treatment (P = 0.008 for medium group and P = 0.043 for thick group), the thin group showed no significant visual improvement (P = 0.141). CRT decreased significantly after treatment in all three groups (P ≤ 0.046) (Fig 4).

### Factors associated with visual function

Factors associated with visual function was statistically analyzed in all eyes without dividing by groups. In Pearson’s correlation coefficient test, baseline BCVA (LogMAR) was negatively correlated with SFCT (rho = -0.254, P = 0.025) but not with age (rho = 0.208, P = 0.068), even if age and SFCT showed strong negative correlation (rho = -0.484, P<0.001). When age was controlled, correlation between BCVA and SFCT was not statistically significant (rho = -0.180, P = 0.118). In multiple linear regression analyses, BCVA at baseline was correlated with SE and SFCT, but not with age (Table 4.). There were no significant factors related with difference between baseline BCVA and 3-month follow-up BCVA by multiple regression analysis.

**Table 4:** Associated factors with visual acuity at baseline (Multivariate analysis).

Baseline Factors	BCVA at baseline (LogMAR)	
	B ± SE	P value
SE	0.088 ± 0.034	0.011
SFCT	-0.001 ± 0.060	0.009

B = Coefficients; SE = standard error

## Discussion

Our results reveal that eyes with PCV have a wide range of choroidal thickness. The thinnest SFCT was 60 μm, and the thickest SFCT was 602 μm. In fact, a previous study showed the SFCT range of 88.0–604.0 μm in PCV eyes, supporting our results. However, the range of SFCT was various in other PCV studies, probably because there were differences in number of study eyes, and in clinical features of study subjects, including age and myopic status. In this study, the number of study eyes were larger than many previous studies, and eyes with high myopia were excluded. The mean age of subjects were early seventies not higher than many previous studies, which were between early sixties and late seventies (S1 Table).[11–14,21–24] Although the range of age was wide (53–91 years), the age of subject who had the thinnest SFCT was 78 years, and the age of subject who had the thickest SFCT was 66 years. Thus, we think that the SFCT range was not accidentally influenced by any skewed distribution of baseline characteristics in study subjects.

The mean SFCT of PCV eyes in this study was almost similar to those of normal healthy eyes, which were among 261.93 ± 88.42 μm,[25] 272 ± 81 μm,[26] 297.8 ± 82.2 μm,[27] and 354 ± 111 μm.[28] However, the SD of SFCT in PCV eyes was larger than those of normal healthy eyes, which means the range of SFCT was wider in PCV eyes. These findings suggest that a simple SFCT measurement in an individual PCV eye may not be useful to distinguish between PCV and other retinochoroidal disorders including AMD CNV. It was suggested that pachychoroid features beneath the neovascular process should be considered as one of the phenotypic manifestations for the diagnostic criteria for PCV.[29] However, as shown in this study, the measurement of SFCT is thin in some PCV eyes, which may induce confusion in the diagnosis of PCV.

In this study, we used mean and SD of SFCT values acquired from 78 eyes with PCV to define the thin, medium, and thick choroid groups. One previous study used mean and SD of normal healthy eyes to define three choroid thickness groups of PCV eyes.[16] However, the SFCT distribution profile in eyes with PCV appeared to be significantly different from that in healthy eyes. Mean SFCT of eyes with PCV could be similar or higher than that of normal healthy eyes, and SD of SFCT in eyes with PCV could be larger than that of normal healthy eyes [12,14,21,23]. Thus, it appears that the method used in this study is more appropriate for PCV eyes than the method used in a previous study.

PCV eyes with a thin choroid were associated with older patients, lower BCVA and a higher frequency of fundus tessellation, than those of eyes with medium or thick choroids. Considering age, fundus tessellation, and the SFCT value previously suggested as a cut-off value for age-related choroidal atrophy (125 μm),[20] more than a half of eyes with PCV and a thin choroid have age-related choroidal atrophy. We suppose that age-related choroidal atrophy accompanied by PCV might decrease retinal function, which is associated with a low BCVA. However, we cannot exclude the possible influence of non-age-related or physiological changes in the choroidal thickness on the visual function, because statistical analysis showed that baseline SFCT, but not age, was associated with baseline BCVA. Further studies are needed in the future.

The data showed that 3-month anti-VEGF treatment improved CRT significantly in all three thickness group. Although multivariate analysis could not show any statistically significant factors associated with visual improvement between baseline and 3 month follow-up, eyes with PCV and a thin choroid had significantly less visual improvement than eyes with PCV and a medium or thick choroid after short-term anti-VEGF treatment. We think that special caution should be given to patients with PCV and a thin choroid, until additional studies reveal the association between thin choroid and visual prognosis after anti-VEGF treatment. One previous study investigated the association between choroid thickness and response to intravitreal ranibizumab treatment in patients with PCV.[16] They reported that, unlike our results, no intergroup difference was found in the functional outcome (visual acuity) in patients with PCV. However, they used different cut-off values to classify choroid thickness (<177, 177–340, and > 340 μm) in the eyes with PCV. Considering that the mean SFCT of the eyes with a thin choroid in that study was 151.6 ± 24.2 μm, many of those eyes would have been classified as medium thickness if our cut-off values were used. Recently, another study reported that baseline greater SFCT was associated with a better visual outcome in PCV eyes.[30] Although we cannot directly compare the result with current data due to different study design and treatment methods, two studies revealed that choroidal thickness might be associated with visual prognosis after treatment. Further prospective, longitudinal studies with larger sample size are needed to assess the influence of choroidal thickness on the visual outcome after the treatment.

It was reported that some of large PCVs extended to the major vascular arcade were reported to have characteristics of neovascularization rather than choroidal vasculopathy. Such eyes usually had polypoidal dilations at the margin of mesh-like vessels, and might show a rapid progression.[31] To minimize the possible influence on SFCT by AMD CNV having polypoidal dilations, we excluded such large PCV in this study.

The current study had several limitations. First, this was a retrospective study, and some patients were excluded due to other treatment or incomplete treatment. Second, we analyzed eyes treated with either bevacizumab or ranibizumab. Thus, it is possible that the two drugs produced a different treatment effect in the eyes with PCV. However, a previous study reported that the short-term response of these two drugs is similar in eyes with PCV.[32] Third, we only analyzed the short-term response to the anti-VEGF treatment, because it was more closely associated with baseline characteristics than the long-term response. Further studies to investigate the long-term treatment response, including polyp closure rate, recurrence rate, and the effect of other treatment modalities are needed. Fourth, only a part of study eyes were included for the treatment response analysis. Although some baseline clinical features of treated eyes were similar to those of total study eyes, studies using larger number of eyes are needed in the future. Fifth, the presence of diurnal variation in choroidal thickness was reported in healthy subjects. Although all measurements were performed at daytime, SFCT was not measured at fixed time of the day in every visit. Thus, diurnal variation of choroidal thickness could affect the results to some extent. Sixth, only SFCT was used as the representative value for choroidal thickness in this study. Recently, new devices and software algorithms providing automated measurements of multiple points, or regions, and wide area of choroidal thickness are developed. We think that multiple point measurements or choroidal volume measurements might enable more accurate assessment in the future investigation.

## Conclusion

Eyes with PCV can not only have a very thick choroid, but also a very thin choroid. The current study revealed that the range of SFCT in PCV eyes was broad. Furthermore, eyes with PCV and a thin choroid outside one SD have different clinical features from those with a medium or thick choroid, as they tend to be associated with older age and show less visual improvement after the initial three consecutive anti-VEGF treatments.

## Supporting information

S1 Table Age of patients and subfoveal choroidal thickness (SFCT) in recent polypoidal choroidal vasculopathy studies. (DOCX)

S1 Appendix Clinical characteristics and ocular measurements data of all subjects. (XLSX)
