# Supplementary material for: Comparison of clinical features and 3-month treatment response among three different choroidal thickness groups in polypoidal choroidal vasculopathy
Source: PLoS One. 2017 Sep 8;12(9):e0184058. doi: 10.1371/journal.pone.0184058 (PMC5590886; doi:10.1371/journal.pone.0184058)
Supplement: S1 Table — (DOCX) [file pone.0184058.s001.docx]

S1 Table. Age of patients and subfoveal choroidal thickness(SFCT) in recent polypoidal choroidal vasculopathy studies.

|  | Number of eyes | Mean age (years) | Mean SFCT (µm) | SFCT range (µm) |
| --- | --- | --- | --- | --- |
| (Chung et al. 2011) | 25 | 68.5 ± 7.1 | 438.3 ± 87.8 | 260-590 |
| (Koizumi et al. 2011) | 25 | 71.7 ± 6.3 | 293.4 ± 73.1 | 128-404 |
| (Jirarattanasopa et al. 2012) | 65 | 73.3 ± 8.0 | 243.3 ± 92.9 | N/A |
| (Yang et al. 2013) | 18 | 63.2 ± 8.3 | 338 ± 10.7 | N/A |
| (Kim et al. 2013) | 40 | 70.3 ± 6.9 | 345.9 ± 113.0 | N/A |
| (Maruko et al. 2013) | 27 | 74.1 ± 8.8 | 231.9 ± 109.2 | 88-604 |
| (Nishide et al. 2013) | 45 | 70.4 ± 8.0 | 243.2 ± 65.6 | N/A |
| (Hikichi et al. 2014) | 86 | 77 ± 8 | 271 ± 102 | N/A |
| (Shin et al. 2015) | 61 | 71.0 ± 7.7 | 326.03 ± 99.65 | N/A |
